# Entities inside one another ‐ a matryoshka doll in biology?

**DOI:** 10.1111/1758-2229.12716

**Published:** 2018-12-26

**Authors:** Tanja Woyke, Frederik Schulz

**Affiliations:** ^1^ U.S. Department of Energy Joint Genome Institute Walnut Creek, CA 94598 USA

Matryoshka dolls, more commonly known as Russian or babushka dolls, are a set of wooden figures nested into each other. The outer doll often displays a female wearing a safaran, a long, traditional Russian folk costume likely dating back to the 14th century. Inside it, the dolls may be male or female, with the smallest of them typically being a baby made out of solid wood. One cannot help but think about matryoshka dolls when looking at biology, and in particular, oddities such as the mealybug symbiosis (Husnik and McCutcheon, 2016; Szabo *et al*., 2017), where a symbiont is contained within a symbiont residing in yet another cell. Previous Crystal Balls have nicely discussed symbiosis from different angles and we do not intend to rehash any of these fascinating discussions and viewpoints. Instead, we provide a perspective on the biological entities (cells and/or particles) physically contained within single microbial cells. Endosymbiosis, in the sense of endocytobiosis, is the most intimate form of symbiosis, as defined by one symbiotic partner (endosymbiont) being housed intracellularly within the second symbiotic partner (host).

It is theorized that symbiogenesis (endosymbiont theory) was a prominent event in the evolution of the eukaryotic cell through the acquisition of an alphaproteobacterium by a proto‐eukaryote, leading to the evolution of the endosymbiont mitochondria (Zimorski *et al*., 2014; Archibald, 2015; Martin *et al*., 2015; Roger *et al*., 2017; Eme *et al*., 2018). Later on in a mitochondrion‐containing eukaryote, cyanobacteria were engulfed and evolved to form chloroplasts (Archibald, 2015; Martin *et al*., 2015). This engulfment of one cell by another cell, or so‐called primary endosymbiosis, is not the end of the story and that is where the matryoshka dolls come into play again. In another theory termed secondary endosymbiosis, resulting products of primary endosymbiosis are then engulfed by a free‐living eukaryotic cell. Such engulfment has occurred several times during the evolution of eukaryotes (Zimorski *et al*., 2014; Archibald, 2015; Martin *et al*., 2015; Gentil *et al*., 2017). Within both green and red algae, for example, secondary endosymbiosis gave rise to secondary plastids. Tertiary endosymbioses, the engulfment of an alga containing a secondary plastid, has been shown in dinoflagellates (Bhattacharya *et al*., 2004).

**Figure 1:**
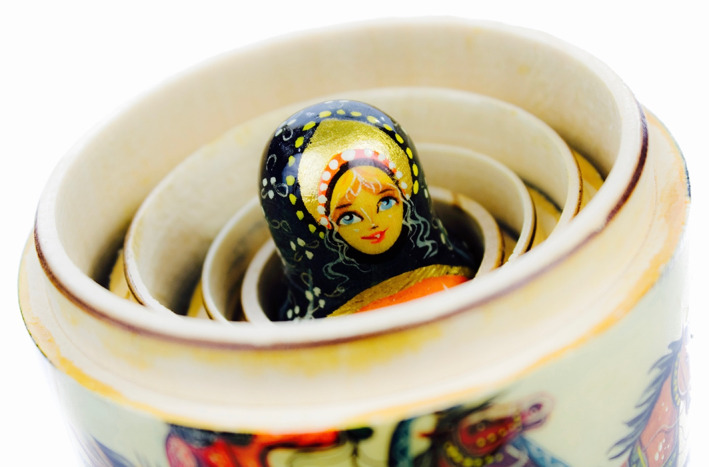
Matryoshka dolls, more commonly known as Russian or babushka dolls, are a set of wooden figures nested into each other. This ‘nested doll’ principle comes to mind when pondering about endosymbiosis in biology.

In addition to these rather ancient engulfment events, there are more recent endosymbiotic associations across the tree of life. In protists, endosymbiosis has facilitated the host's acquisition of expanded metabolic functions for increased biochemical versatility (Nowack and Melkonian, 2010). As such, protist hosts have acquired functions including photosynthesis, nitrogen fixation, methanogenesis and sulfide oxidation through their symbiotic partnerships. A prominent example is the cercozoan amoeba *Paulinella chromatophore*, which associated with cyanobacterial ancestors to sustain its phototrophic lifestyle (Bhattacharya *et al*., 2004). However, the nature of most endosymbiotic interactions remains elusive. The intracellular niches that are occupied by endosymbionts are manifold; some examples include the nucleus (Schulz and Horn, 2015), the perinuclear space (Schulz *et al*., 2015), the endoplasmic reticulum (Vogt, 1992), chloroplasts (Wilcox, 1986) and mitochondria (Sassera *et al*., 2006; Deeg *et al*., 2018). In particular, the latter two are great examples for the Matryoshka doll principle, as symbionts acquired relatively recently replicate inside symbionts acquired hundreds of millions of years ago (Fig. 1).

A fascinating example of an endosymbiont retained inside of a bacterium is the parasite of *Thiotrix* (Larkin *et al*., 1990). In the early 1990s, imaging data enabled the discovery of bacterial parasitic endosymbionts of the sulfide‐oxidizing gammaproteobacterium *Thiothrix*; these endosymbionts were contained within either the periplasm or cytoplasm. Surprisingly, nearly 30 years after this discovery, very little information and evidence exists for modern endosymbioses between two non‐eukaryotic cells. The few examples that do exist indicate that bacteria are able to successfully enter another bacterial host cell. Considering billions of years of evolution of bacterial cells, which would provide ample time to form stable associations through cell engulfment, this apparent lack of endosymbioses without eukaryotic involvement is astonishing.

While the terminology of endosymbiosis applies to non‐viral associations only, we also want to discuss other nucleic acid containing intracellular entities, specifically large DNA viruses. Giant nucleocytoplasmic large DNA viruses (NCLDV) were discovered approximately 15 years ago (La Scola *et al*., 2003) and have been found to infect a diversity of eukaryotes, particularly protists and algae. These viral giants are defined by their large particle sizes (>/= 200 nm) and genome sizes (at least 200–300 kb) and can themselves be infected by viruses called ‘virophages’ (Claverie and Abergel, 2016; Fischer, 2016). The recent discovery of a giant virus genome from a wastewater treatment plant strongly supports the hypothesis that giant viruses evolved from smaller viruses by taking up bits and pieces of DNA from their hosts (Schulz *et al*., 2017). More recent metagenome studies further show that the currently assessed diversity of giant viruses is likely a vast underestimate (Schulz *et al*., 2018; Mihara *et al*., 2018).

Although engulfments that led to endosymbiosis have drastically shaped the evolutionary history of life on Earth, surprisingly few have been discovered. Looking into the Crystal Ball, we foresee discovering that these ‘entities inside one another’ are much more prevalent than one might envision and previously appreciated. These will be identified by mining and re‐mining of global sequence datasets, future cost‐effective long‐read sequencing technologies and advanced higher throughput single‐cell as well as imaging and (co‐)cultivation approaches. In addition, a more accurate picture of the diversity of intracellular entities will be revealed, as we have already witnessed through recent big data mining efforts (Paez‐Espino *et al*., 2016; Roux *et al*., 2016; Alberti *et al*., 2017) yet more sophisticated experimental work will go beyond computational predictions to begin connecting symbiont to host. Several single‐cell genomics studies of microorganisms have already linked viruses to their hosts (Yoon *et al*., 2011; Roux *et al*., 2014; Roy *et al*., 2014; Labonte *et al*., 2015) and have the potential to identify possible endosymbionts.

The paltry evidence for modern bacterial/archaeal endosymbioses is surprising. If these are more prevalent than current evidence suggests, most cultivation efforts may have failed to recover them. If they were present in sequencing projects, they may have been discarded as ‘contamination’ during the quality control and assembly processes. Genome‐resolved metagenomics would likely fail to connect them, as they would be split into different genome bins. Considering that many branches in the bacterial and archaeal tree of life are solely represented by metagenome assembled genomes, and to a smaller extent by single amplified genomes, many of which believed to depend on metabolic partners (Castelle *et al*., 2015; Baker *et al*., 2017; Castelle and Banfield, 2018) or even shown intracellularly within a host (Gong *et al*., 2014), there are numerous evolutionary paths yet to be explored for such associations.

Superficially, endosymbioses are akin to matryoshka dolls. But once we look closely at the intimate, intricate and diverse multipartite relationships that have evolved over hundreds of millions of years, we realize that these associations between cells (and viruses) are more than just simple stacking. During the early stages of endosymbiosis, these once independent evolutionary trajectories must adapt to survive and reproduce together as a new organism. In our Crystal Ball, we see the use and continued development of advanced tools that will accelerate discovery and characterization of these fascinating associations.

While one cannot go back in time, we can use the information of modern life to reconstruct the most likely past. Finding more examples of stable engulfments will shed light on the metabolic interplay between the partners, including their communication and the level of host control. This will broaden our understanding of how intracellular entities co‐evolved with their hosts to occupy and adapt to new environmental and energetic niches, which led to the staggering diversity of life we observe today.
